# Enzymatic hydrolysis of *Chlorella sorokiniana* biomass using fungal enzyme mixtures: screening, optimization, and proteomic analysis for enhanced microalgal biomass valorization

**DOI:** 10.1007/s00449-026-03356-0

**Published:** 2026-06-06

**Authors:** Timo Dräger, Annika Krimmel, Felix Melcher, Michael Paper, Melania Pilz, Daniel Garbe, Norbert Mehlmer, Corinna Dawid, Thomas Brück

**Affiliations:** 1 Werner Siemens-Chair of Synthetic Biotechnology, TUM School of Natural Sciences, Technical University of Munich, Garching, Germany; 2 Chair of Food Chemistry and Molecular Sensory Science, TUM School of Life Sciences, Technical University of Munich, Freising, Germany; 3 ZIEL – Institute for Food & Health, Technical University of Munich, Freising, Germany; 4 Professorship for Chemosensory Food Systems, TUM School of Life Sciences, Technical University of Munich, Freising, Germany; 5 Leibniz Institute for Food Systems Biology at the Technical University of Munich, Freising, Germany

**Keywords:** *Chlorella sorokiniana*, Enzymatic hydrolysis, *Aspergillus awamori*, Cell wall degradation, Proteomics, Microalgal biomass valorization

## Abstract

The sustainable production of microalgal biomass for food and feed applications requires efficient downstream processing methods, particularly for the disruption of recalcitrant microalgal cell walls. This study aimed to develop an enzymatic hydrolysis method using fungal enzyme mixtures for effective degradation of the cell wall of the green microalga *Chlorella sorokiniana*. Eight fungal strains were screened for their enzyme production and hydrolysis efficiency, with *Aspergillus awamori*,* Aspergillus niger*, and *Ceratocystis paradoxa* showing the highest performance and selected for detailed analysis. A Design of Experiment approach optimized hydrolysis parameters, revealing that all enzyme mixtures showed maximal sugar release at 60 °C and an enzyme-to-biomass ratio of 5% (v/w). Among these, *A. awamori* enzyme mixtures exhibited the highest sugar-to-protein ratio, indicating superior catalytic efficiency. Proteomic analysis highlighted distinct enzymatic profiles between *A. awamori* and a commercial reference enzyme preparation (Cellic CTec3), with *A. awamori* showing higher glucosidase, mannosidase, and chitinase activities, which correlated with enhanced hydrolysis of chitin-like polysaccharides in the microalgal cell wall. These findings suggest that enzyme mixtures tailored to the specific carbohydrate composition of microalgae, which combine cellulolytic, mannosidic, and chitinolytic activities, can enhance microalgal biomass hydrolysis efficiency. Such enzyme mixtures represent promising candidates for sustainable bioconversion and valorization of *C. sorokiniana* biomass in industrial bioprocesses.

**Supplementary Information:**

The online version contains supplementary material available at https://doi.org/10.1007/s00449-026-03356-0.

## Introduction

The global food demand is projected to increase by 50% by the year 2050 due to a rapidly growing world population [1–3]. With food production accounting for approximately 34% of anthropogenic greenhouse gas emissions, alternative solutions beyond traditional agriculture and livestock are urgently needed to ensure long-term food security [4]. Microalgae are becoming increasingly important in the food industry as a sustainable, nutrient-dense alternative to conventional food sources.

They possess a high protein content with a favorable essential amino acid profile and exhibit a lipid profile rich in polyunsaturated fatty acids. In addition, microalgae are a source of other bioactive compounds and dietary components such as vitamins and antioxidants, making them a valuable addition to a healthy diet [5–9]. Moreover, microalgae have several advantages in comparison to terrestrial crops, including rapid growth rates, high photosynthetic efficiency, and CO_2_ fixation ability. Additionally, as unicellular organisms, they lack non-photosynthetic structures and need fewer structural polysaccharides (e.g., cellulose), resulting in higher photosynthetic efficiency and higher productivity than crops.

Microalgae can also be harvested frequently and are not limited by growth and maturation cycles like crops, limiting the possible number of harvests per year. The productivity per space is therefore significantly higher for microalgae cultivation than for conventional cultivation of crops [10]. To this end, microalgae cultivation requires reduced land, water, as well as energy and can therefore be carried out on non-arable land, like urban environments, potentially reducing the environmental footprint of food production and increasing food security [8, 11–13]. Moreover, the possibility of cultivating microalgae using brackish or seawater presents an additional advantage, avoiding competition with freshwater resources. Microalgae cultivation can be done solely through photosynthetic growth, resulting in a potentially carbon-neutral footprint and addressing critical challenges in the food and feed industry. This makes producing microalgal biomass a highly promising solution for sustainable food and feed applications [14, 15].

*Chlorella sorokiniana* is a small freshwater, unicellular, green microalga with a cell diameter of 2–4.5 μm. *C. sorokiniana* is capable of mixotrophic growth on different carbon and nitrogen sources, as well as fast phototrophic growth with doubling times reported as low as 4–6 h, making this species ideal for industrial applications [7]. *C. sorokiniana* is also capable of growth in wastewater under conditions that are unfavorable for other microalgae, such as carbon dioxide supplementation from flue gas and cultivation in higher temperatures [16–18]. Besides the favorable growth characteristics *C. sorokiniana* has a high nutritional value, particularly its protein content, essential amino acids, vitamins, minerals and beneficial phytochemicals [19–21].

However, one major challenge lies in the efficient and cost-effective downstream processing of microalgae, especially the cell disruption to obtain intracellular compounds, such as proteins, lipids, pigments, and bioactive compounds [22]. An efficient and mild cell wall degradation is therefore a step to obtaining the intracellular compounds without the risk of their alteration or degradation. There are different cell disruption methods, which are generally classified into mechanical and non-mechanical methods [23–26].

Mechanical methods like high-pressure homogenization and bead milling show excellent disruption capabilities, often resulting in complete disintegration of microalgal biomass [24, 25, 27]. However, these methods require a high energy input per biomass unit, resulting in an energy-intensive method to obtain intracellular compounds [26]. Additionally, the harsh conditions employed to crack microalgal cell walls can damage or degrade sensitive intracellular compounds, reducing product quality and yield [22, 23].

In contrast to the mechanical cell disruption and chemical hydrolysis often resulted in high salt concentrations and performed using harsh conditions, enzymatic cell lysis holds many advantages over other cell disruption methods, such as mild operating conditions, low energy requirements, low capital investment, and the prevention of harsh physical conditions [23]. However, enzymatic treatments require a detailed understanding of the microalgal cell wall composition, which can vary significantly across different microalgal species [28, 29]. As a result, the effectiveness of enzymatic lysis largely depends on the compatibility between the enzyme and the specific cell wall structure. Consequently, selecting appropriate enzymes is a critical parameter influencing the disruption efficiency [23].

Enzymatic disruption of microalgal cell walls offers a selective alternative to mechanical methods, with promising results but dependent on the species or even cultivation conditions like nitrogen limitation [30–34]. Enzymes effectively weaken or rupture cell walls to enhance intracellular product recovery, though high costs and long reaction times limit scalability [31–33]. The methane yield from biogas production could be increased by 20% using *Chlorella sorokiniana* biomass, using an additional enzymatic pretreatment. In addition, glucans of the cell wall from *Chlorella homospaera* could be efficiently hydrolyzed using a mix of endoglucanase and β-glucosidase on untreated biomass. Optimized hydrolysis of *Scenedesmus* sp. improved lipid extraction, while tailored cocktails disrupted *Chlorella vulgaris* walls without compromising lipid stability. Immobilized multienzyme systems also achieved high sugar/protein yields from consortia [31, 32, 35].

Limitations include incomplete lysis of resistant species like *Nannochloropsis* sp., suboptimal commercial cocktails for microalgal cell walls, slow kinetics, enzyme inhibition, and high dosages [32, 33, 36]. Recent advances, such as novel glucosidase enzyme mixtures and ultrasound-assisted hydrolysis, show strong potential alongside autolysis and immobilization, thus demonstrating that enzymes excel as hybrid biorefinery tools [27, 31–34].

In recent years, fungi have been used to produce a broad range of secreted enzymes in large amounts covering more than 50% of the global enzyme market due to their low production cost, high yield, and simplified downstream processing using simple, established purification techniques [37, 38]. A significant share of the enzyme market is dominated by a few species, namely *Aspergillus*, *Trichoderma*, *Rhizopus*, and *Penicillium*, which can generate enzymes at commercially-viable scales [37]. While, depending on the production process, the commercially available enzyme mixtures commonly do not contain specific hydrolases for microalgae cell walls, the production of specific enzyme mixtures using the biomass itself as nutrient source for fungi cultivation can significantly increase the hydrolytic efficiency [39].

This work aimed to develop an efficient and simple enzymatic method for cell wall hydrolysis of the microalga *Chlorella sorokiniana*. Therefore, eight fungal strains were initially screened for enzyme production and the hydrolysis efficiency of *Chlorella sorokiniana* biomass based on enzyme activity assays. To induce the production of specific enzyme mixture containing the activities needed for efficient hydrolysis, the fungi strains were cultivated on the biomass as sole nutrient source. Three out of these eight strains were selected for subsequent hydrolysis experiments. These strains were further analyzed using proteomics to identify the enzyme activities and assess the efficiency of different enzyme compositions in microalgal cell wall hydrolysis.

## Materials and methods

*Chlorella sorokiniana* powder was supplied by Aliga microalgae (Aliga ApS, Hjørring, Denmark). The microalgae were grown under heterotrophic conditions in controlled stainless steel bioreactors, subsequently harvested, and spray-dried [40] (Fig. S1).

### Fungal strains and media

Eight different fungal strains were selected for the following cultivation and hydrolysis experiments. The individual strains were selected based on enzyme activity assays, specifically qualitative screenings for amylolytic, lignocellulolytic, and proteolytic enzyme activities as described by Barišić [41].

The selected fungal strains are listed in Table 1, with their abbreviations used further on.

**Table 1:** Fungi strains (name and strain) selected for the cultivation and hydrolysis experiments, selected based on enzyme activity assays with and their abbreviations

Fungi species	Strain	Abbreviation used
*Ceratocystis paradoxa*	CBS 374.83	*C. paradoxa_CBS*
*Ceratocystis paradoxa*	DSM 63,054	*C. paradoxa_DSM*
*Trichoderma reesei*	RUT C30	*T. reesei*
*Aspergillus awamori*	DSM 63,272	*A. awamori*
*Aspergillus niger* van Tieghem	ATCC 10,535	*A. niger*
*Aspergillus terreus*	CBS 117.37	*A. terreus_1*
*Aspergillus terreus*	CBS 594.65	*A. terreus_2*
*Aspergillus terreus*	CBS 116,686	*A. terreus_3*

Algae-agar plates were prepared consisting of 1% (w/v) agar and microalgae powder in 1%, 2% or 3% (w/v) concentrations. All plates were inoculated with a small piece of mycelium in the centre of the plate and were incubated at 28 °C for 7 days. After 7 days, the growth of mycelium and the possible formation of a hydrolysis halo around fungus cultures were analyzed.

Liquid media consisting of water with microalgal biomass concentrations of 2%, 3%, 4% or 5% (w/v) as the sole nutrient source were prepared. As positive controls, potato extract [0.4% (w/v)] and glucose [3% (w/v)] were added to the algae-containing media. All cultivations were done in 100 ml cell culture flasks with a membrane-containing ventilation cap (Sarstedt AG & Co. KG, Nümbrecht, Germany). The flasks were inoculated with small pieces of mycelium from the previous agar plates and were cultivated at 28 °C and 110 rpm for 7 days.

The best-growing fungal strains were subsequently cultivated in 5 l baffled flasks. Each flask contained 2 l biomass suspension in water (3% (w/v)) and was inoculated with 400 ml of a 7-day-old fungi pre-culture. The subsequent cultivation lasted 14 days and was carried out at 28 °C and 110 rpm.

### Enzyme mixture processing

The collected culture supernatants were filtered through cotton wool and progressively smaller pore-size bottle-top filters up to 0.2 μm (Sarstedt AG & Co. KG, Nümbrecht, Germany). The enzyme containing culture medium was exchanged to a 0.1 M phosphate buffer (pH 6) and subsequently concentrated to a final volume of about 45 ml using a Pall Minimate tangential flow filtration (TFF) system equipped with an Omega 10 kDa TFF Capsule (Cytiva, Marlborough, USA). The enzyme mixtures were stored at + 4 °C until further usage. Phosphate buffer was selected due to its broad pH range, with the pH adjusted at pH 6 to accommodate that most fungal cellulolytic enzymes show pH optima around 4–6 [42, 43].

### Chemical hydrolysis

As a reference for the examined enzymatic treatments and their efficiency in hydrolyzing the microalgal cell wall, a chemical hydrolysis of the used *Chlorella sorokiniana* powder was adapted from Melcher et al. [40]. Microalgal biomass (10 mg/ml) was incubated in water at 95 °C for one hour. Following centrifugation at 19,000 rcf for 10 min at room temperature. The supernatant and pellet were separated, and both were acidified with concentrated sulfuric acid, resulting in a final concentration of 2% (v/v). The acidified supernatant and pellet were autoclaved at 121 °C for one hour. Solid calcium carbonate was used to neutralize the solutions after autoclaving. Precipitated calcium salts and other insoluble components were removed by centrifugation immediately after the neutralization. 2 ml of each supernatant was collected and frozen at –20 °C overnight. After thawing, the samples were filtered through 0.2 μm syringe filters (Phenomenex LTD, Aschaffenburg, Germany) and were used for sugar measurements. Chemical hydrolysis was done in biological triplicate.

### Design of experiment experimental setup

The Design of Experiment (DoE) experimental plans were created with the software DESIGNEXPERT, Version 23.1.8 (2023 Stat-Ease, Inc., Minneapolis, MN). As the response factor, the released sugar concentrations in mg/ml were chosen with the goal of maximizing the sugar concentrations. As variables, the temperature (range 40–60 °C), the pH (range 5.5–8), and the enzyme-to-biomass ratio (range 0.5–5% (v/w)) were set up. As the underlying model, a response surface design was chosen, which evaluates all main effects, two-factor interactions, and quadratic terms. For each enzyme mixture, an experimental plan with 16 individual runs was created by the software. Each run was done in biological triplicate, and the arithmetic means were used in further calculations.

### Enzymatic hydrolysis

Enzymatic hydrolysis was done in biological triplicate. All experiments had an algal biomass concentration of 1% (w/v) suspended in 100 mM phosphate buffer, with the pH adjusted. Two enzyme-to-biomass ratios of 0.5% (v/w) and 5% (v/w) were tested for each enzyme mixture. The algal suspension was incubated with the enzyme mixtures for 72 h at 60 °C under constant shaking at 120 rpm. Sugar concentrations before and after the addition of the enzymes were measured, and the released sugars due to the hydrolysis of the present enzymes were calculated by the difference between these measurements. The sugar concentrations were also corrected by the amount of sugars in control runs without enzyme addition.

### Sugar measurements via HPLC

An aliquot of the sample was filtered using 10 kDa centrifugal filters (VWR, Darmstadt, Germany). 197 µl of the filtrate and 3 µl of 0.5 M EDTA were added to an HPLC vial. The monomeric sugar mixtures were separated using a Agilent Infinity II 1260 HPLC according to Jurkowski et al. [44] using an isocratic separation with 5 mM sulfuric acid at a flow rate of 0.5 ml/min at 70 °C on a Rezex ROA-Organic Acid H+ (8%) ion-exclusion column (300 mm, 7.8 internal diameter; Phenomenex LTD, Aschaffenburg, Germany). Chemical hydrolysis samples were measured with a run time of 60 min, and enzymatic hydrolysis samples with a run time of 40 min.

### Protein concentration measurements

The protein concentration was measured with a bicinchoninic acid (BCA) assay (Sigma-Aldrich, Taufkirchen, Germany) in 96-well plates following the vendor’s instructions. Briefly, 25 µl of standard or sample was added to an individual well of a 96-well plate. 200 µl of BCA working reagent was added to each well and mixed for 30 s on a plate shaker. The plate was incubated at 37 °C for 30 min and cooled afterwards to room temperature. Then, the absorbance at 562 nm was measured. Samples were diluted to fit in the calibration range of 0 to 1000 µg/ml.

### Proteomics

The purified and concentrated enzyme mixtures (see 2.4) were prepared and analyzed by means of a proteomics approach to determine the present hydrolytic enzyme activities.

#### Sample preparation

Proteome analysis was done in technical triplicates for each fungal enzyme mixture as described by Arnold et al. [45]. 10 µl of fungal enzyme mixture were mixed with 40 µl Laemmli buffer and incubated at 95 °C for 5 min [46]. Afterwards, the samples were loaded on an SDS-PAGE (stacking gel and separation gel with 5% (w/v) and 12% (w/v) acrylamide, respectively) for separation. The electrophoresis was stopped as soon as the samples reached the separation gel. The loaded gels were stained with Coomassie Brilliant Blue dye and subsequently destained using a solution consisting of 5% (v/v) isopropanol, 5% (v/v) acetic acid, and 50% (v/v) ethanol in water. The stained protein bands were excised from the gels, cut into small pieces (< 1 mm^3^), washed, dried under a vacuum for at least 15 min, and subsequently used for peptide isolation.

The dried samples were resuspended in 100 µl 10 mM dithiothreitol solution and incubated at 56 °C for 45 min at a shaking speed of 550 rpm. The samples were washed with acetonitrile, and 100 µl of iodoacetamide solution (55 mM) were added. Afterwards, the samples were incubated at room temperature in the dark for 30 min at 550 rpm. Then, the samples were washed twice with acetonitrile and 50 mM ammonium bicarbonate (1:1 (v: v)) and dried under vacuum for at least 15 min. Subsequently, the samples were resuspended in 100 µl of trypsin digestion solution, containing 1 µl trypsin solution in 100 µl 25 mM ammonium bicarbonate solution. The tryptic digestion was carried out overnight at 37 °C in a thermal shaker at 300 rpm. Afterwards, 100 µl of 25 mM ammonium bicarbonate solution was added, and the samples were sonicated for 15 min. Then, 100 µl of acetonitrile and sonication for 15 min were added. The peptide-containing supernatants were transferred to a separate collection tube. 100 µl 5% (v/v) formic acid was added to the gel pieces, and the samples were sonicated for 15 min. After adding 100 µl of acetonitrile and another sonication, the supernatant was collected and added to the collection tube. This step was repeated twice. The liquids from the collection tube were evaporated under vacuum for at least 2.5 h. The dried peptide samples were resuspended in 20 µl 1% (v/v) formic acid, sonicated for 10 min, and filtered through 10 kDa centrifugal filters to remove undigested protein fragments. The filtered samples were subsequently analyzed using LC-MS/MS.

#### LC-MS/MS

Peptide analysis was done using an LC-MS/MS analysis with a timsTOF Pro mass spectrometer equipped with a NanoElute LC System (Bruker Daltonik GmbH, Bremen, Germany) on an Aurora column (250 × 0.075 mm, 1.6 μm; IonOpticks). The mobile phase comprised a 0.1% (v/v) water–formic acid mixture (A) and a 0.1% (v/v) acetonitrile–formic acid mixture (B), which was added as a binary gradient at a flow rate of 0.4 µl/min. The gradient started at 2% (v/v) B and linearly increased to 17% B after 36 min. After 54 min, the gradient was increased to 25% (v/v) B and linearly to 37% (v/v) B after 60 min. After 70 min, the concentration of B was adjusted to the final value of 95% (v/v). The oven temperature during the measurements was 50 °C. The timsTOF Pro mass spectrometer (TIMS) was used in PASEF mode with the following settings: mass range: 100–1700 mass: charge [m/z] ratio; ion mobility ramp, 0.6–1.6 V s/cm^2^; 10 MS/MS scans per ion mobility ramp (total cycle time 1.16 s); charge range, 0–5; active exclusion for 0.4 min; a target intensity of 20,000 counts; and an intensity threshold of 1,000 counts. The collision energy was ramped stepwise, appropriate to the ion‐mobility ramp, from 20 to 59 eV. The electrospray ionization source parameters were 1,600 V for the capillary voltage and 3 l/min N_2_ (as dry gas) at a dry gas temperature of 180 °C. The measurements were performed in a positive ion mode. Mass calibration was performed using the sodium formate cluster, and the TIMS was calibrated using Hexais (2,2-difluoroethoxy) phosphazene, Hexakis (2,2,3,3‐tetrafluoropropoxy) phosphazene, and Chip cube high mass references (m/z ratios of 622, 922, and 1222, respectively).

#### Bioinformatics

The MS/MS tandem spectrometry data of tryptic-digested peptides were evaluated using the PEAKS studio software (v.10.6, build 20201221). The annotated genome of *Aspergillus awamori* and *Trichoderma reesei* Rut C30 served as a reference for protein identification [47, 48]. The database search parameters included a precursor mass of 25 ppm, employing monoisotopic mass, and a fragment ion error tolerance of 0.05 Da. Trypsin was used as the digestion enzyme, allowing a maximum of two missed cleavages per peptide. Up to three variable PTMs were allowed per peptide, and FDR estimation was performed using decoy fusion. A 1.0% FDR threshold was applied for protein identification, requiring at least one unique peptide per protein. The PEAKSQ software was used to identify the different enzymes in each sample group, consisting of technical triplicates for each. A mass error tolerance of 20.0 ppm, an ion mobility tolerance of 0.05 Da, and a retention time shift tolerance of 6 min (auto detect) were employed.

The relative expression level of each selected enzyme was calculated by dividing its expression level by the total expression level of all selected enzymes in the experiment. The expression levels were represented as the area calculated by the PEAKS studio software. Activities sharing the same EC number were then combined.

$$\begin{aligned}&\text{Relative expression strength } [{\%}]\\ & \quad =\frac{\text{Area for individual enzyme}}{\text{Sum of the area of all individual enzymes}}\end{aligned}$$

## Results

### Fungi cultivation

**Fig. 1:**
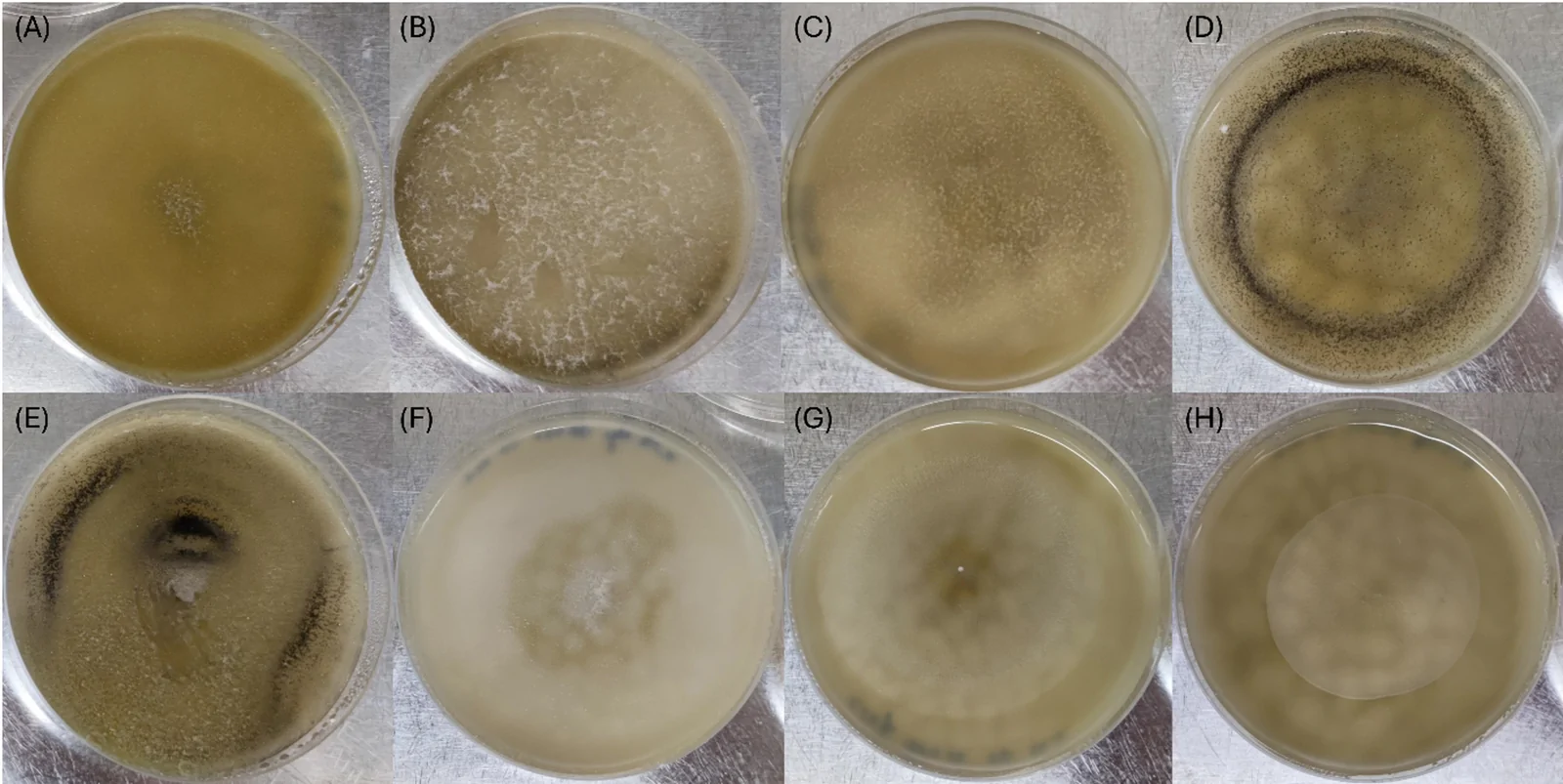
Algae agar plate (0.5% (v/w) microalgae powder); cultivation for 7 days at 28 °C; **A** *Ceratocystis paradoxa* CBS 374.83; **B** *Ceratocystis paradoxa* DSM 63,054; **C** *Trichoderma reesei* RUT C30; **D** *Aspergillus awamori* DSM 63,272; **E** *Aspergillus niger* van Tieghem ATCC 10,535; **F** *Aspergillus terreus* CBS 117.37; **G** *Aspergillus terreus* CBS 594.65; **H** *Aspergillus terreus* CBS 116,686

After 7 days of incubation, mycelium growth in different amounts was observed for all examined fungal strains (Fig. 1; S8–S13). Figure 1 shows algae-agar plates containing 0.5% (w/v) microalgae powder after 7 days with fungal growth of the eight tested strains. Mycelial growth and discoloration of the agar plate are visible on the plate shown, suggesting that fungal growth is possible if microalgal biomass is directly used as the sole nutrient source.

Cultivation in liquid cultivation demonstrated that the growth was sustained through the decomposition of the microalgae and not the agar used for the plates (Fig. S14-S16). In liquid cultures, mycelial growth was visible after 7 days of cultivation at 28 °C and 110 rpm in most cultures. Most notable were the fungal strains *A. awamori* and *A. niger*, which showed the most mycelial growth in combination with the incorporation of the whole microalgal biomass content in their mycelium, resulting in a clear supernatant free of microalgae.

The three strains *Ceratocystis paradoxa* DSM 63,054, *Aspergillus awamori* DSM 63,272, and *Aspergillus niger* van Tieghem ATCC 10,535 out of the eight tested fungal strains were selected for further cultivation based on the visible mycelial growth and incorporation of microalgal biomass.

### Characterization of the fungal enzyme mixture

The fungal enzyme mixtures obtained were characterized regarding their optimized incubation parameters, protein concentration, cellulase activity, hydrolysis efficiency on microalgae, and proteomics.

#### Optimization of the hydrolysis parameters

**Fig. 2:**
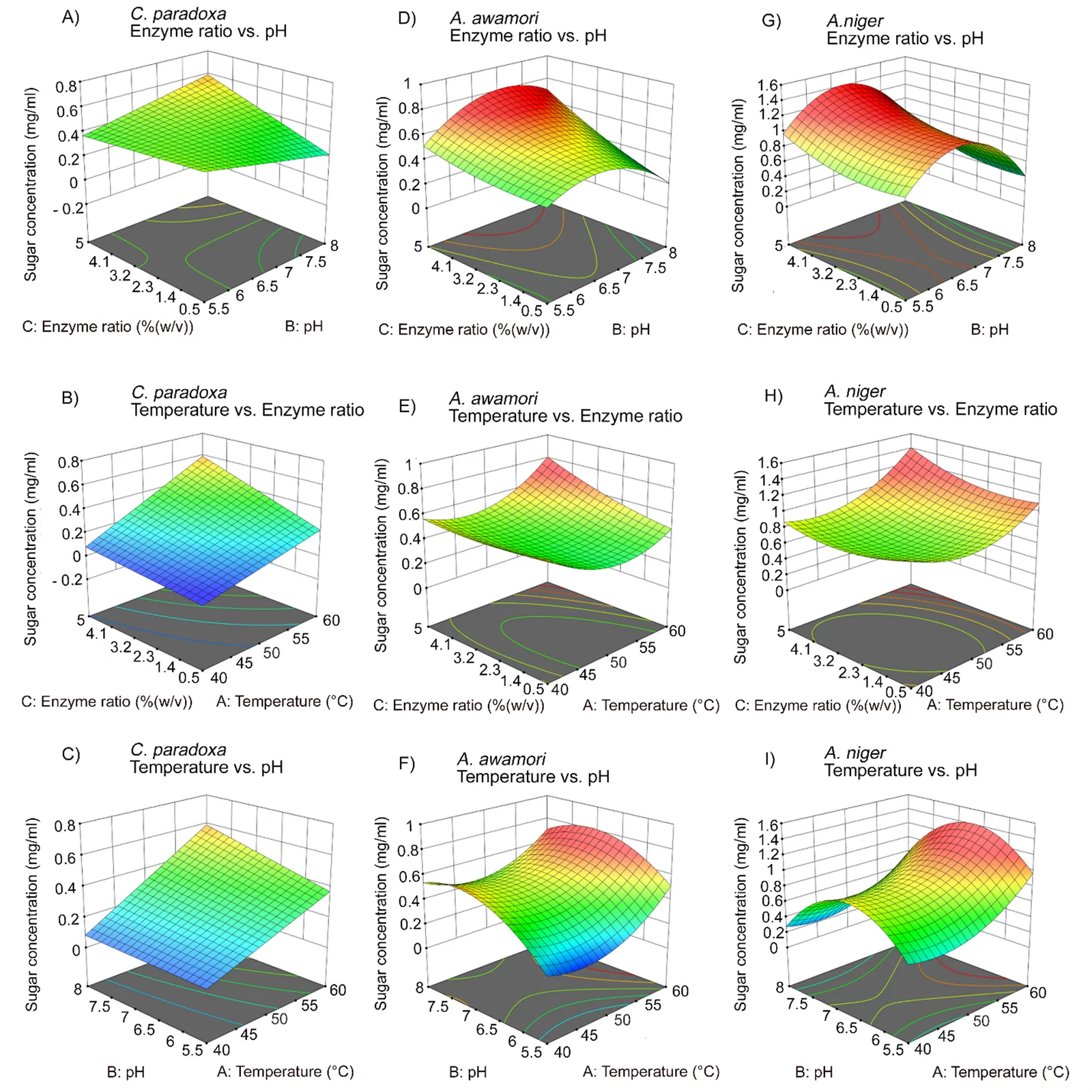
Results of design of experiment parameter optimization for fungal supernatants; **A** *C. paradoxa* enzyme ratio vs. pH; **B** *C. paradoxa* temperature vs. enzyme ratio; **C** *C. paradoxa* temperature vs. pH; **D** *A. awamori* enzyme ratio vs. pH; **E** *A. awamori* temperature vs. enzyme ratio; **F** *A. awamori* temperature vs. pH; **G** *A. niger* enzyme ratio vs. pH; **H** *A. niger* temperature vs. enzyme ratio; **I** *A. niger* temperature vs. pH

A Design of Experiment (DoE) approach was chosen to screen for the optimized hydrolysis parameters for each fungal enzyme mixture. All runs were done in biological triplicate, and the arithmetic means of the total released sugar concentrations were used as the response factor. Figure 2 shows each enzyme mixture’s analysis graphs and optimal parameter settings.

Table 2 shows the optimal parameter settings for each enzyme mixture. All enzyme mixtures showed the highest sugar release at 60 °C and an enzyme-to-biomass ratio of 5% (v/w). After a set of validation experiments, the total sugar concentrations were measured to review the expected sugar concentrations calculated from the DoE. Only *A. awamori* could confirm the expected sugar concentrations, with an expected amount of 0.815 mg/ml and a measured concentration of 0.796 mg/ml, resulting in a deviation of 2.3%. For *C. paradoxa* and *A. niger*, the measured sugar concentrations were lower than the expected amounts, resulting in a deviation of 42.5% and 48.5%, respectively.

**Table 2:** Expected released sugar concentrations for the optimized parameter settings calculated from the Design of Experiment and the measured released sugar concentrations from the confirmation experiment

Fungal strain	Temperature [°C]	pH	Enzyme ratio [%(v/w)]	Expected sugar concentration [mg/ml]	Measured sugar concentration [mg/ml]
*C. paradoxa*	60	7.6	5	0.709	0.408
*A. awamori*	60	7.1	5	0.815	0.796
*A. niger*	60	6.6	5	1.35	0.695

#### Hydrolysis efficiency

**Fig. 3:**
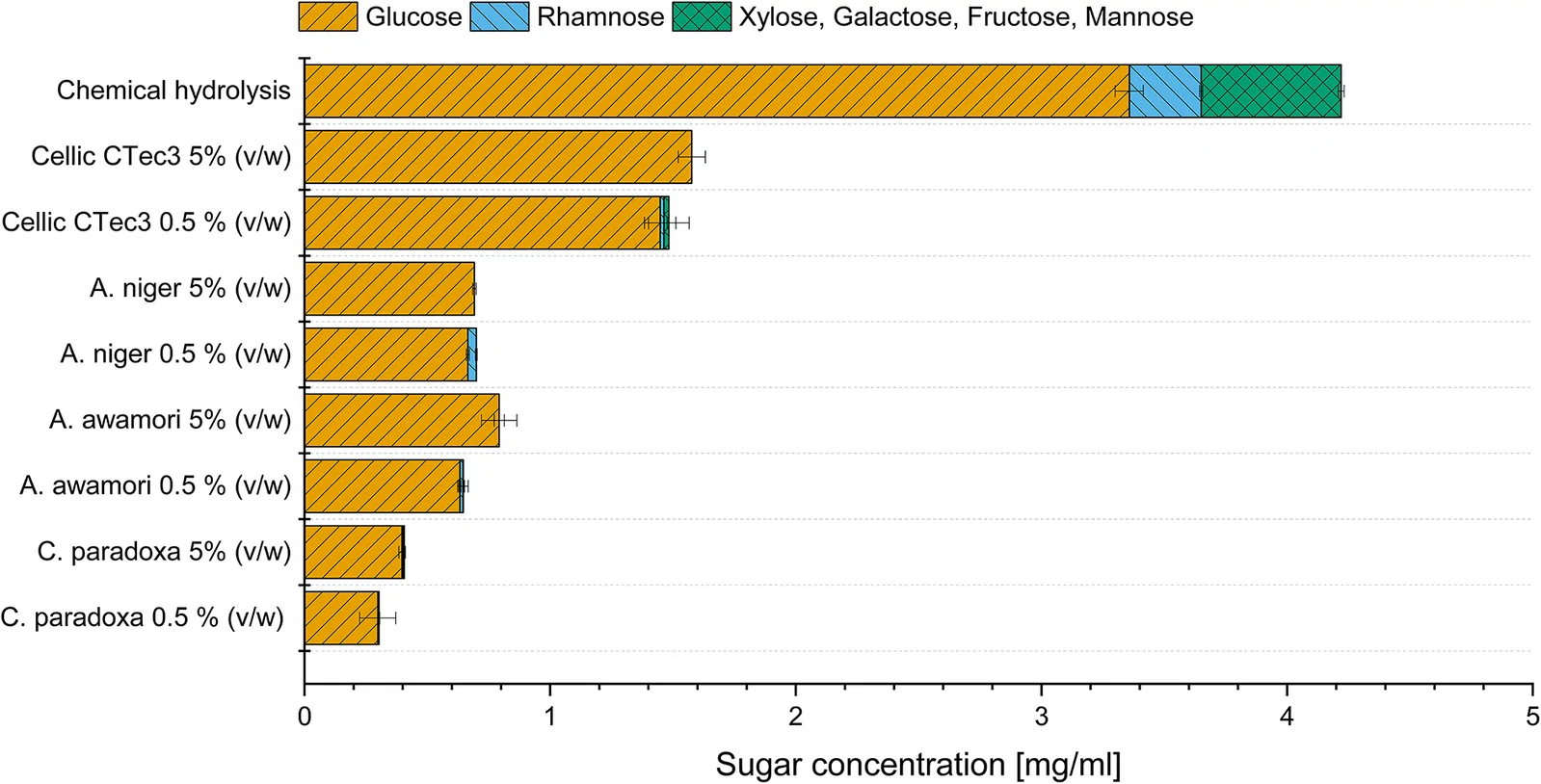
Released sugar concentrations after 72 h incubation with enzyme mixtures at their respective pH- and temperature-optima; total sugar and sugar composition chemical hydrolysis experiments for each condition were conducted as triplicates, and the arithmetic means are shown and their respective standard deviations

Hydrolysis experiments were set up to determine and compare the hydrolysis efficiency of the enzyme mixtures produced in this work. All experiments (fungal enzyme mixtures and Cellic CTec3) were performed using an algal biomass concentration of 1% (w/v) and an enzyme-to-biomass ratio of 0.5% and 5% (v/w). Microalgae were suspended in 100 mM phosphate buffer, and the pH was adjusted to their respective optimized value. The mixtures were incubated for 72 h at their optimal temperature under constant shaking. Sugar concentrations were compared before and after incubation; the difference represents the amount of released sugar due to the hydrolysis of the present microalgal biomass. As a reference for sugar release, chemical hydrolysis was performed as described in 2.3. The total sugar concentration consisted of 3.36 mg glucose/ml, 0.293 mg rhamnose/ml and 0.57 mg xylose, galactose, fructose and mannose/ml, resulting in a total sugar concentration of 4.22 mg/ml (Fig. 3).

**Fig. 4:**
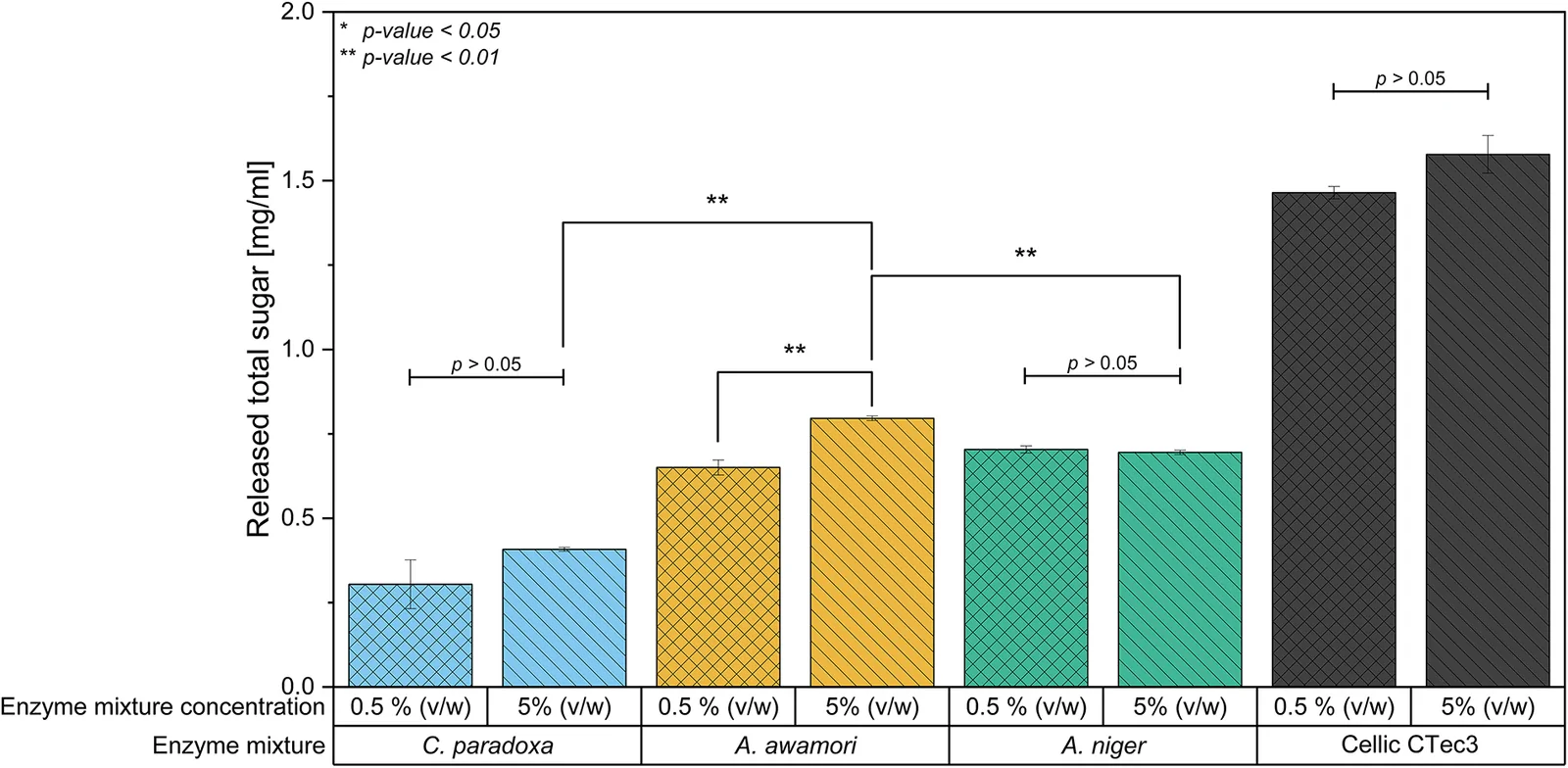
Released sugar concentrations after 72 h incubation with enzyme mixtures at their respective pH- and temperature-optima; experiments for each condition were conducted as triplicates, and the arithmetic means are shown and their respective standard deviations

Comparing the sugar release to the other self-produced mixtures, the enzyme mixture obtained from *A. awamori* showed the highest concentration of released sugars among the self-produced enzyme mixtures, with 0.8 mg/ml at an enzyme ratio of 5% (v/w), showing a significant increase in sugar concentrations compared to an enzyme ratio of 0.5% (v/w) with 0.65 mg/ml (Fig. 4). Cellic CTec3, used as commercially available reference, showed the highest sugar release with 1.57 mg/ml at an enzyme-to-biomass ratio of 5% (v/w), almost 2-fold higher than the highest self-produced enzyme mixture from *A. awamori* at an enzyme ratio of 5% (v/w) with 0.8 mg/ml. Compared to the total sugar concentration after chemical hydrolysis of 4.22 mg/ml, Cellic CTec3 released 35.2% and 37.4% of the total sugars. The highest fungal enzyme mixture was *A. awamori* with 15.4% and 19%, followed by *A. niger* with 16.7% and 16.5%. The lowest release could be seen with the *C. paradoxa* enzyme mixture at 7.1% and 9.7%.

#### Protein content

To investigate if different protein concentrations could be the reason for the difference in sugar release, the protein concentrations, and therefore the enzyme concentrations, were measured using a bicinchoninic acid (BCA) assay. Each enzyme mixture was diluted 1:100, and Cellic CTec3 was diluted 1:400 to obtain measurements in the calibration range of 0 to 1000 µg protein/ml.

**Fig. 5:**
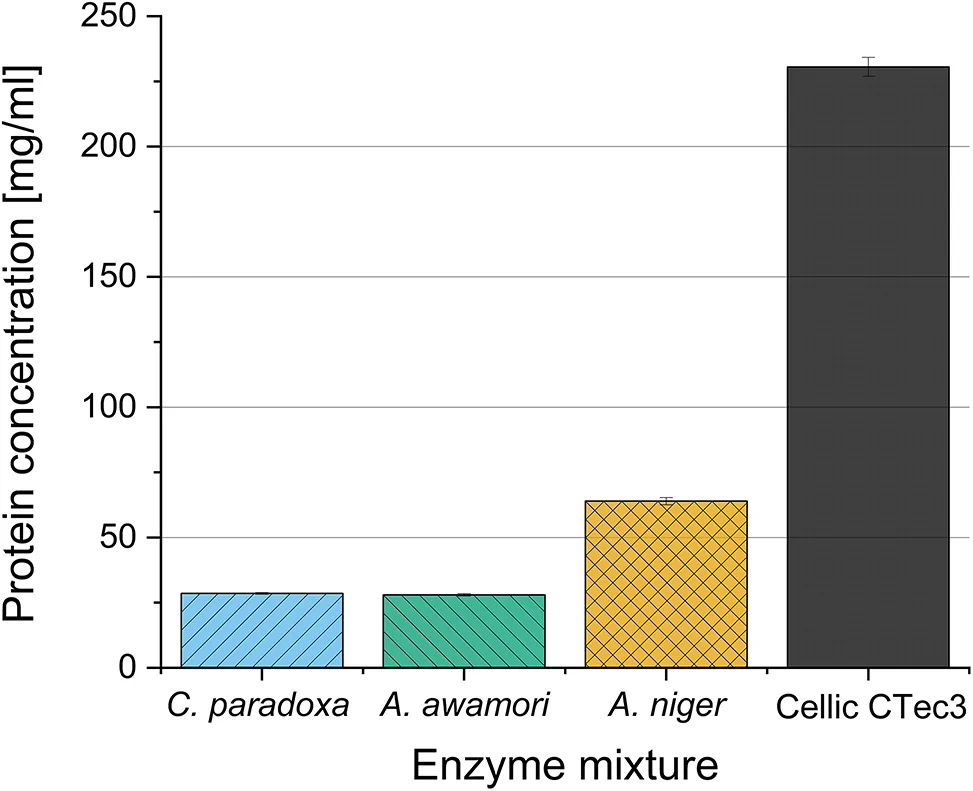
Protein concentrations [mg/ml] of different fungal enzyme mixtures and Cellic CTec3 measured with BCA assay; Fungal enzyme mixtures were diluted 1:100, and Cellic CTec3 was diluted 1:400

The enzyme mixture collected from *A. niger* showed the highest protein concentration of the fungal enzyme mixtures, with 63.95 mg/ml. Enzyme mixtures collected from *C. paradoxa* and *A. awamori* contained similar protein concentrations of 28.54 mg/ml and 28.02 mg/ml, respectively. Cellic CTec3 showed a significantly higher protein concentration of 230.6 mg/ml. Therefore, it contains more than 3.5 times and 8 times more protein than the fungal enzyme mixtures (Fig. 5).

As the significant difference in protein concentrations between the fungal enzyme mixture and Cellic CTec3 can be a reason for the different absolute sugar release concentrations, the released sugar concentrations were divided by the respective protein concentration to determine how much sugar was released per amount of protein.

**Fig. 6:**
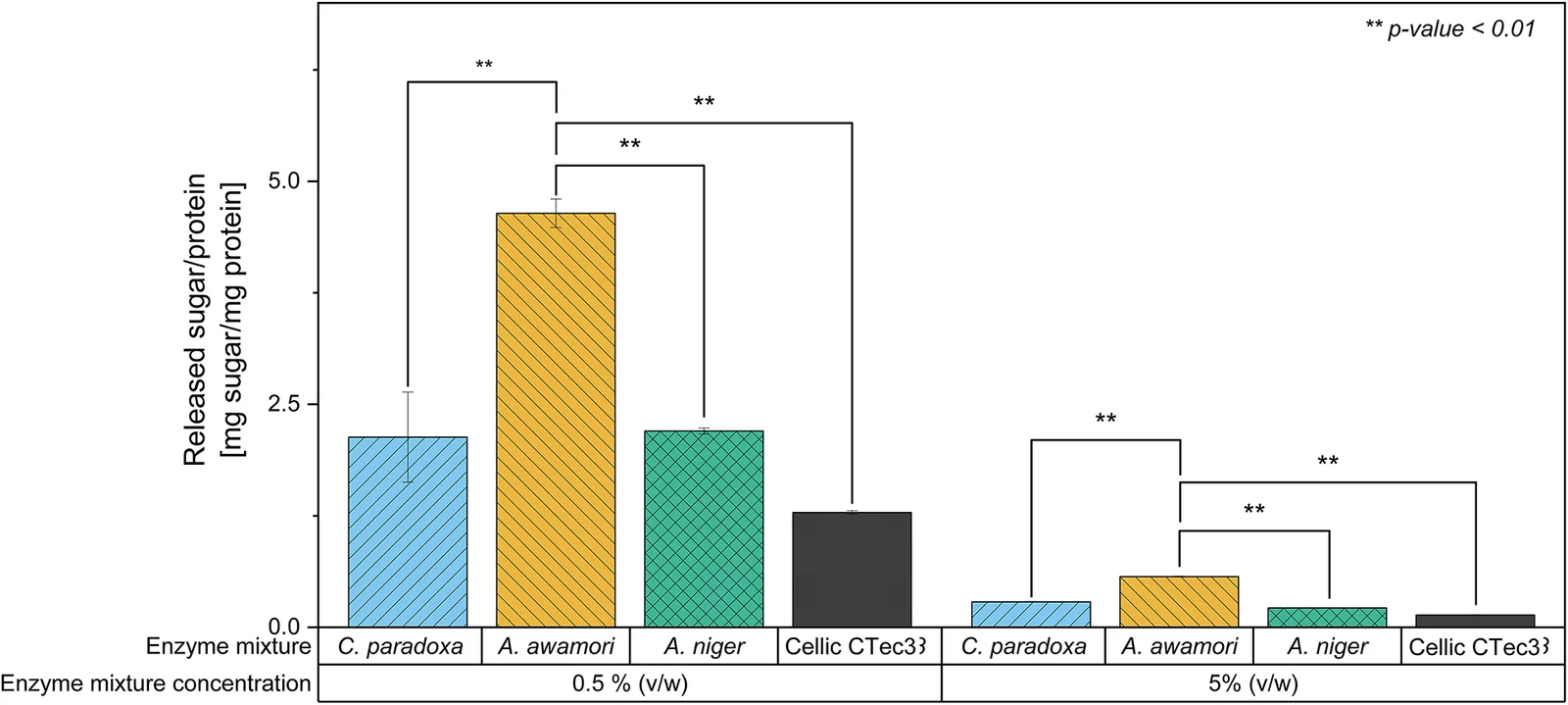
Released sugar-to-protein ratios in mg sugar/mg protein after 72 h incubation with enzyme mixtures at their respective pH- and temperature-optima; experiments for each condition were conducted as triplicates, and the arithmetic means are shown and their respective standard deviations; grouped by the enzyme ratios; brackets and asterisks symbolizing the significance between to shown samples

*A. awamori* showed the highest sugar-to-protein ratio of 4.64 mg sugar/mg protein, significantly higher than for all other samples within the same sample set (Fig. 6). The difference in the ratio between the 0.5% (v/w) and 5% (v/w) samples was significantly higher at 0.5% (v/w) for all mixtures. Most notable is that the lowest sugar-to-protein ratio in both sample sets (0.5% v/w and 5% v/w) is for Cellic CTec3 with 1.28 mg/mg and 0.14 mg/mg for 0.5% (v/w) and 5% (v/w), respectively. On the other hand, comparing Cellic CTec3 at 0.5% and the mixtures from *C. paradoxa*, *A. awamori* and *A. niger* at 5% to have more comparable total amount of protein, Cellic CTec3 shows a higher sugar to protein ration than the other enzyme mixtures.

#### Proteomics

The enzyme mixture from *A. awamori* exhibited the highest hydrolysis efficiency when normalized to its protein concentration and was therefore selected for comparative proteomics analysis to gain insight into the enzyme activities present and responsible for its improved performance. Cellic CTec 3, a commercial enzyme formulation, showing the highest overall sugar release, was used for comparison. Although the specific strain is not known, it is documented that the filamentous fungi *Trichoderma reesei* is used in the production of Cellic CTec 3 [49–51].

Only enzymes with a -10lgP score above 150 – indicating statistically reliable identifications - were included. The − 10lgP scores were calculated with the PEAKS studio software. The relative expression level of each selected enzyme was calculated by dividing its expression level by the total expression level of all selected enzymes in the experiment. Activities sharing the same EC number were then combined [52].

Proteomic data were filtered to focus on enzymes with hydrolytic activity toward carbohydrates. Proteases were excluded from the following, after reviewing their expression strengths. In *A. awamori*, no protease activity could be found with a relative expression strength over 0.00%, for Cellic CTec3 endoprotease activity could be detected with a relative expression strength of 0.32%.

**Fig. 7:**
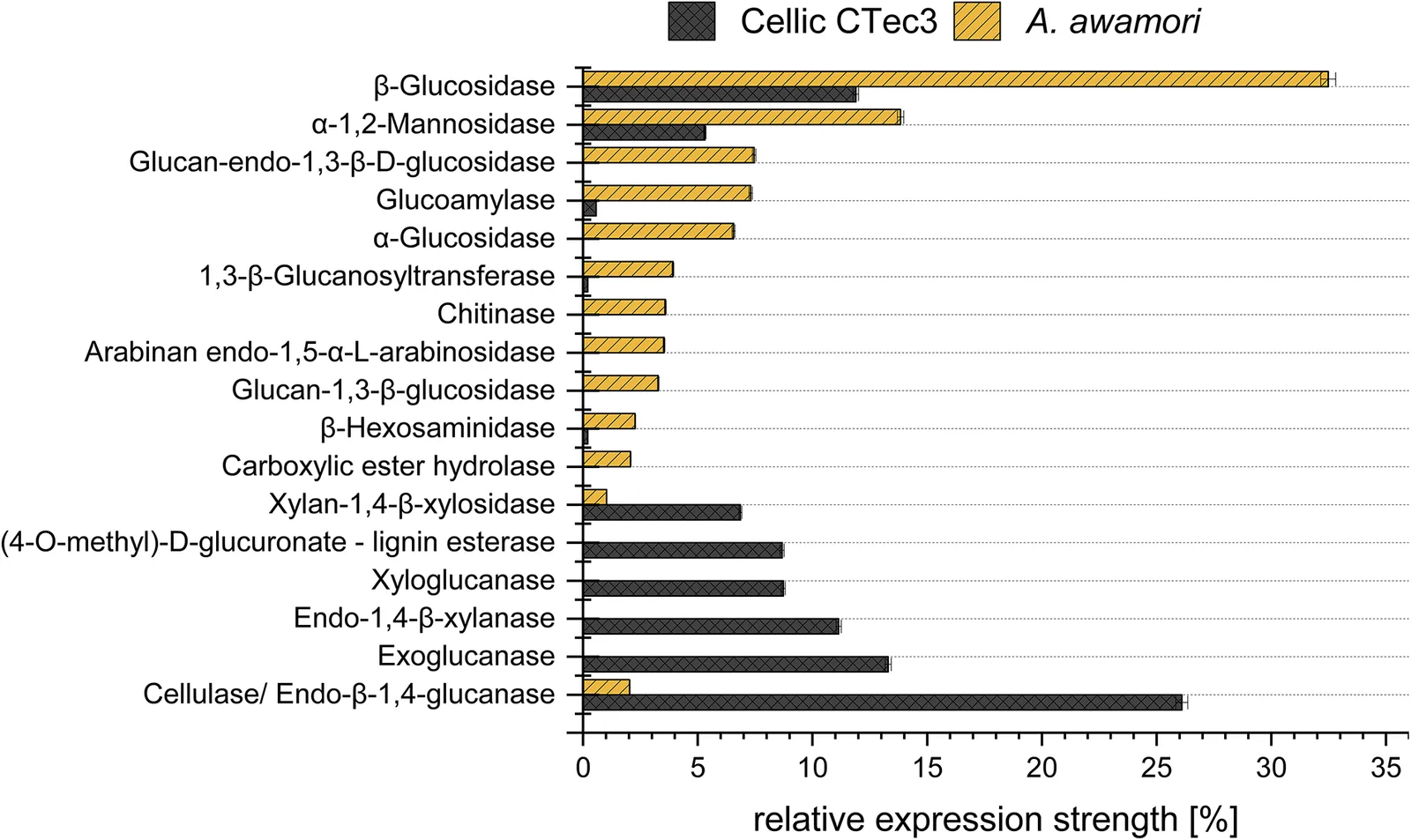
Relative expression strength [%] based on individual enzymes for *A. awamori* and Cellic CTec3 and their respective standard deviations

The relative expression strength for enzymes above 2% in at least one sample was determined for *A. awamori* and Cellic CTec3 (Fig. 7, Tab. S1). While Cellic CTec3 showed the highest expression for cellulase/Endo-β-1,4-glucanase (26.1%), exoglucanase (13.3%) and β-glucosidase (11.89%). *A. awamori* exhibited the highest values for β-glucosidase (32.48%), α-1,2-mannosidase (13.84%) and glucan-endo-1,3-β-D-glucosidase (7.46%).

Of the 17 enzyme activities assessed, only seven were present in both mixtures. The β-glucosidase activity was the most abundant one present in both samples, accounting for 32.48% in *A. awamori* and 11.89% in Cellic CTec3. Notable differences were observed for cellulases, exoglucanase and endo-1,4-β-xylanase, which were considerably higher in Cellic CTec3, and for β-glucosidase, which was more prominent in *A. awamori*. Chitinase activity was only detected in *A. awamori* (3.59%).

**Fig. 8:**
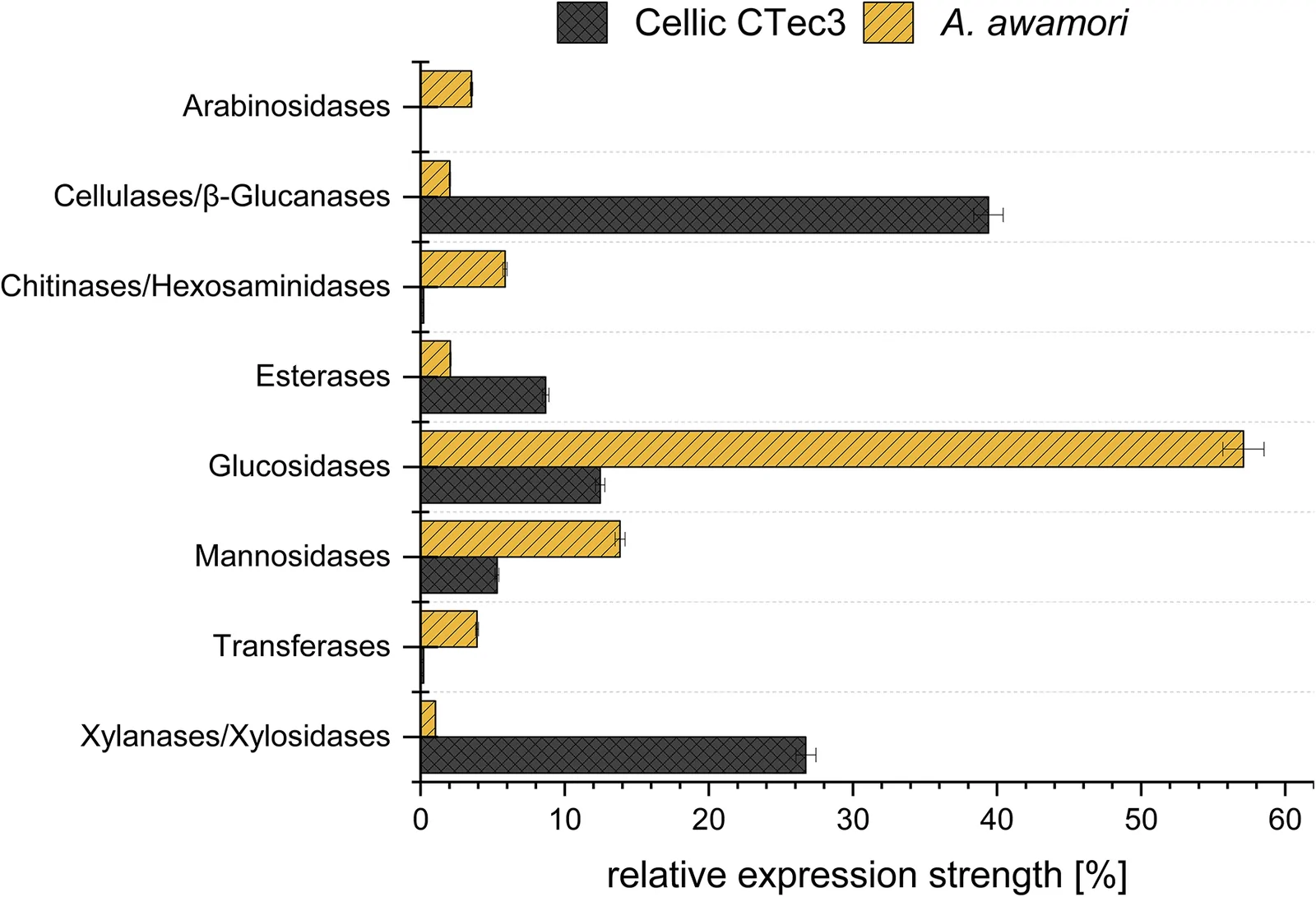
Relative expression strength [%] for different enzyme clusters and their respective standard deviations; clusters were based on the substrate specificity and/or cleavage point

Grouping the detected enzymes highlights distinct enzymatic profiles. *A. awamori* had the strongest overall representation of glucosidases (57.09%), compared to 12.46% in Cellic CTec3. Conversely, the cellulase/β-glucanase group showed the highest expression in Cellic CTec3 with 39.04%, and only 2.03% in *A. awamori*. Similarly, xylanases/xylosidases were much more abundant in Cellic CTec3 (26.73%) than in *A. awamori* (1.03%), while mannosidases and chitinases/hexosaminidases activities were higher in *A. awamori* (Fig. 8).

Other carbohydrates, such as starch, can also be present in microalgal cells, influencing the enzyme expression. *Chlorella sorokiniana* is a known producer of starch under certain cultivation conditions; therefore, amylase activities were additionally considered [53–55]. In Cellic CTec3, no amylase activities could be detected. In the *A. awamori* enzyme mixture, a relative expression strength of 0.84% was detected for amylase activity, indicating some degree of starch degradation. Nonetheless, a relative expression strength of 0.84% is relatively low compared to the other enzyme activities detected in the *A. awamori* enzyme mixture.

## Discussion

The aim of the study was to find a suitable fungal enzyme mixture for breaking down the cell wall of *Chlorella sorokiniana*. A selection of eight filamentous fungal strains of the phylum Ascomycota, including *Ceratocystis paradoxa* (CBS 374.83 and DSM 63054), *Trichoderma reesei* (RUT C30), *Aspergillus awamori* (DSM 63272), *Aspergillus niger* van Tiegheim (ATCC 10535) and *Aspergillus terreus* (CBS 117.37, CBS 594.65 and CBS 116686) were chosen with the intention of covering a broad and diverse enzymatic activity spectrum.

*Trichoderma reesei* is considered the leading organism for industrial cellulase production and provides highly characterized and efficient cellulase and hemicellulase profiles [56]. *Aspergillus* species were selected due to their broad spectrum of glycoside hydrolases (e.g. xylanases, glucanases, β-glucosidases, glucoamylases, pectinases) and high pH tolerance, which make them ideal for the breakdown of complex carbohydrates in agricultural residues [57].

That includes *Aspergillus niger*, which is considered to be one of the most significant industrial production organisms, predominantly due to its utilization as a citric acid producer, but also because of its capacity to produce high levels of secretory enzymes. Furthermore, *A. niger* is generally recognized as safe (GRAS) for the production of citric acid [58].

Due to its saccharifying enzymes and its ability to produce citric acid, *Aspergillus awamori* has mainly been used for the production of “honkaku shochu” a traditional Japanese single distilled liquor [59]. *Aspergillus terreus* is also of industrial interest, in addition to its use in itaconic acid production, it is known to produce various secondary metabolites, such as lovastatin [60].

*Ceratocystis paradoxa*, a plant pathogen that is relatively unknown among the other fungi, is characterized by its high enzyme expression and differential utilization of various carbons sources, such as lignocellulosic material [61, 62]. The findings of the present study demonstrated that all fungal strains selected for this study have the capacity to utilize *Chlorella sorokiniana* biomass (0.5% (v/w)) as their sole source of nutrition, which demonstrates the remarkable adaptability of these fungi, as a result of specific enzyme expression.

In particular, the *Aspergillus* strains *A. awamori* (DSM 63272) and *A. niger* van Tieghem (ATCC 10535) were found especially efficient at utilizing algae biomass in the initial direct comparison on agar plates (Fig. 1B). It is not surprising that these two species in particular exhibit similar behavior, as molecular phylogenetic studies, e.g. based on mitochondrial cytochrome b analysis, have shown that *Aspergillus awamori*, also known as *Aspergillus luchuensis*, is genetically very closely related to *Aspergillus niger* [63, 64]. In addition to the two *Aspergillus* strains, *C. paradoxa* also demonstrated comparatively high microalgae biomass hydrolysis efficiency.

Consequently, a DoE approach was utilized to ascertain the optimal incubation parameters for the enzyme mixtures of *A. niger*, *A. awamori* and *C. paradoxa* to further enhance the efficiency of hydrolysis (Fig. 2). For the highest sugar release, which can be considered as an indicator of high enzyme activity, an incubation temperature of 60 °C was found to be the optimum for all three strains.

With regard to pH, the enzyme mixtures of *A. niger* and *A. awamori* reached their maximum at pH 6.6 and 7.1, respectively, while the mixture of *C. paradoxa* was slightly above this at pH 7.6. Previous studies showed comparable temperature optima between 50 and 60 °C for enzymes of *A. niger* and *A. awamori*, whereas the reported pH optimum, ranging from 4 to 6, is slightly lower than the values determined for microalgae biomass [65–67].

In addition to strain-specific differences and varying expression profiles due to different substrates, the buffer capacity of the *Chlorella* biomass solution used could contribute to these deviations and influence enzyme activity and the resulting sugar release. Regarding the ratio of enzyme solution to biomass, all strains exhibited a similar pattern: up to a concentration of approximately 3% (v/w), sugar release remained almost constant, while it increased further above that level, which resulted in the maximum being reached at 5% (v/w) in each case (Fig. 2).

This finding suggests that the complete synergistic hydrolysis cascade of the enzymes employed is only attained when the enzyme quantity is sufficiently high. Cellic CTec3, a commercial enzyme mixture used as a reference in the subsequent hydrolysis efficiency study, demonstrated significantly higher absolute sugar release compared to the fungal enzyme mixtures (Fig. 4), indicating either a higher hydrolytic capacity toward *Chlorella sorokiniana* cell walls or a higher enzyme dosage in the enzyme formulation used. Cellic CTec3 also showed the highest release percentage based on the total sugar concentration determined after chemical hydrolysis, with around 36% [40]. *A awamori* released, as the best fungal enzyme mixture, up to 19%.

To enable a more objective comparison of enzymatic performance, the total protein content of each enzyme preparation was determined. Cellic CTec3 contained 3.5- to 8-fold higher protein concentrations than the self-produced fungal enzyme mixtures (Fig. 5). After normalization to protein content, *A. awamori* mixture showed the highest sugar-to-protein ratio, indicating high hydrolysis efficiency on a protein-specific basis (Fig. 6).

A comparative proteomic analysis of the *A. awamori* enzyme mixture and Cellic CTec3 revealed distinct enzymatic profiles, with only seven of seventeen significant enzyme activities shared between both preparations (Tab. S1). Cellic CTec3 was characterized by high levels of cellulases, xylanases, and xylosidases, which are responsible for its pronounced lignocellulolytic and hemicellulolytic activities. Cellulases hydrolyze 1,4-β-glycosidic linkages in cellulose and related β-glucans, while xylanases and xylosidases cleave 1,4-β-xylosidic linkages in hemicellulosic polymers comprising xylose, arabinose, galactose and mannose residues [68, 69]. These enzyme activities were comparatively weak in the *A. awamori* mixture, indicating a lower ability to degrade lignocellulosic materials.

In contrast, *A. awamori* exhibited high abundances of glucosidases, mannosidases, and especially chitinase and hexosaminidase activities. Glucosidases catalyze the removal of terminal glucose units from glycosidic linkages, whereas mannosidases act on mannose-containing polysaccharides, releasing mannose monomers and thus contributing to hemicellulolytic functionality. The most defining feature of the *A. awamori* preparation was the presence of chitinases and hexosaminidases, both of which cleave *N*-acetylglucosamine-containing polysaccharides but via different catalytic mechanisms. Chitinases catalyze the endo-hydrolysis of *N*-acetyl-β-D-glucosaminide 1,4-β-linkages in chitin, a polymer of *N*-acetylglucosamine units. Hexosaminidases, in contrast, hydrolyze terminal non-reducing *N*-acetyl-D-hexosamine residues in *N*-acetyl-β-D-hexosaminides [70]. In Cellic CTec3, only hexosaminidase activity was detected at trace levels.

This enzymatic discrepancy is particularly relevant in the context of *Chlorella sorokiniana* cell wall composition. Previous studies have described chitin-like or glucosamine-containing polysaccharides as key structural components [22, 71]. The microalgal cell wall consists of a multilayered structure, with an outer sporopollenin layer, a secondary layer comprising mannose and chitin-like polysaccharides, and beneath it, the plasma membrane [71, 72]. Weber et al. identified cellulose and chitin-like polymers contributing to the rigidity and resistance of *Chlorella* sp. cell walls against mechanical and enzymatic disruption [22]. These chitin-like structures are complex heteropolymers that likely consist of both glucosamine and other monosaccharides. The chitinase and hexosaminidase activities observed in the *A. awamori* mixture enable hydrolysis of the N-acetylglucosamine-containing regions of the cell wall, resulting in the release of additional measurable reducing sugars. Other cellulolytic or hemicellulolytic enzymes may then act on the remaining non-glucosamine polysaccharides, facilitating more comprehensive degradation. In contrast, Cellic CTec3, which lacks significant chitinase activity, is unable to efficiently hydrolyze glucosamine-containing linkages, resulting in complete degradation and residual glucosamine-enriched polysaccharide fragments.

The relatively high total protein concentration in Cellic CTec3 accounts for its higher absolute sugar release, despite the absence of chitinolytic activities. The enzyme mixture is optimized for lignocellulosic biomass, and its cellulase and hemicellulase system effectively targets cellulose and hemicellulose polymers, yielding high glucose and other monosaccharide concentrations. The up to 8-fold higher protein concentration of Cellic CTec3 compared to the fungal enzyme preparations increases its apparent hydrolysis yield, as more catalytic units are available for substrate turnover. Nevertheless, the *A. awamori* enzyme mixture, combining cellulolytic, mannosidic, and chitinolytic activities, exhibited higher efficiency per gram protein when applied to microalgal biomass. This indicates that enzyme mixtures adapted to the specific carbohydrate composition of microalgal cell walls - rather than lignocellulosic substrates - may achieve improved bioconversion efficiency. Such tailored cocktails may thus represent a promising bioprocessing approach for future microalgal biomass valorization.

## Conclusion

The fungal enzyme mixtures produced in this study demonstrated significant potential for efficient hydrolysis of *Chlorella sorokiniana* biomass, with *Aspergillus awamori* exhibiting superior catalytic efficiency when normalized to protein content. Key findings revealed that *A. awamori* enzyme profiles were particularly rich in glucosidase, mannosidase, and uniquely chitinase activities. These enzymes target the complex carbohydrate structures characteristic of microalgal cell walls, including chitin-like polysaccharides, enabling more effective degradation than commercial preparations lacking chitinolytic activity. Thus, fungal enzyme mixtures tailored to the specific polysaccharide composition of microalgae offer a promising and sustainable bioprocessing strategy for downstream valorization of microalgal biomass in industrial applications. Further optimization of the enzyme production as well as the enzyme mix itself, already performed iteratively in decades for Cellic CTec3, like product inhibition of the glucosidases or addition of more stable enzymes with the same activities from other species, may increase the overall sugar yield and the economic viability of tailored enzyme mixtures.

## Supplementary Information

Below is the link to the electronic supplementary material.

Supplementary Material 1

## Data Availability

Supplementary material is available online. Additional data is available by email; on reasonable request to the corresponding authors.
