# Supplementary material for: Enzymatic hydrolysis of Chlorella sorokiniana biomass using fungal enzyme mixtures: screening, optimization, and proteomic analysis for enhanced microalgal biomass valorization
Source: Bioprocess Biosyst Eng. 2026 Jun 6;49(9):2507–22. doi: 10.1007/s00449-026-03356-0 (PMC13582154; doi:10.1007/s00449-026-03356-0)
Supplement: Supplementary file 1 [file 449_2026_3356_MOESM1_ESM.docx]

Enzymatic Hydrolysis of *Chlorella sorokiniana* Biomass Using Fungal Enzyme Mixtures: Screening, Optimization, and Proteomic Analysis for Enhanced Microalgal Biomass Valorization

Timo Dräger^1,2,3,4^, Annika Krimmel^1^, Felix Melcher^1^, Michael Paper^1^, Melania Pilz^1^, Daniel Garbe^1^, Norbert Mehlmer^1,‡^, Corinna Dawid^2,3,4,5,‡^, Thomas Brück^1,3,‡^

^1^Werner Siemens-Chair of Synthetic Biotechnology, TUM School of Natural Sciences, Technical University of Munich, Garching, Germany

^2^Chair of Food Chemistry and Molecular Sensory Science, TUM School of Life Sciences, Technical University of Munich, Freising, Germany

^3^ZIEL – Institute for Food & Health, Technical University of Munich, Freising, Germany

^4^Professorship for Chemosensory Food Systems, TUM School of Life Sciences, Technical University of Munich, Freising, Germany

^5^Leibniz Institute for Food Systems Biology at the Technical University of Munich, Freising, Germany

‡ Corresponding authors

Norbert Mehlmer [norbert.mehlmer@tum.de](mailto:norbert.mehlmer@tum.de)

Corinna Dawid [corinna.dawid@tum.de](mailto:corinna.dawid@tum.de)

Thomas Brück [brueck@tum.de](mailto:brueck@tum.de)

**Keywords**

*Chlorella sorokiniana*, enzymatic hydrolysis, *Aspergillus awamori*, cell wall degradation, proteomics, microalgal biomass valorization

**B)**

**A)**

Figure S 1: Microscopy images (A: chlorophyll fluorescence, B: transmitted) of the Chlorella sorokiniana biomass used for the experiments

Figure S 2: DoE-experiments carried out for the enzyme mix from C. paradoxa

Figure S 3: Fit statistics for the resulting model for the enzyme mix from C. paradoxa

Figure S 4: DoE-experiments carried out for the enzyme mix from A. niger

Figure S 5: Fit statistics for the resulting model for the enzyme mix from A. niger

Figure S 6: DoE-experiments carried out for the enzyme mix from A. awamori

Figure S 7: Fit statistics for the resulting model for the enzyme mix from A. awamori

**Table S1** Summarization of the relative expression strength for enzyme activities above 2% in at least one sample, A  awamori or Cellic CTec3. Enzyme activities detected in both samples are shown in bold

| Enzyme name | Relative expression strength [%] | |
| --- | --- | --- |
|  | *A. awamori* | Cellic CTec3 |
| *Glucosidases* |  |  |
|  |  |  |
| Glucan-1,3-β-glucosidase | 3.28 | n.d. |
| Glucan-endo-1,3-β-D-glucosidase | 7.46 | n.d. |
| Glucoamylase | 7.31 | 0.57 |
| α-Glucosidase | 6.56 | n.d. |
| β-Glucosidase | 32.48 | 11.89 |
|  |  |  |
| *Mannosidases* |  |  |
|  |  |  |
| α-1,2-Mannosidase | 13.84 | 5.31 |
|  |  |  |
| *Xylosidases* |  |  |
|  |  |  |
| Endo-1,4-β-xylanase | n.d. | 11.14 |
| Xylan-1,4-β-xylosidase | 1.03 | 6.86 |
|  |  |  |
| *Arabinosidases* |  |  |
|  |  |  |
| Arabinan endo-1,5-α-L-arabinosidase | 3.53 | n.d. |
|  |  |  |
| *Chitinase/Hexosaminidases* |  |  |
|  |  |  |
| Chitinase | 3.59 | n.d. |
| β-Hexosaminidase | 2.27 | 0.21 |
|  |  |  |
| *Cellulase/β-Glucanases* |  |  |
|  |  |  |
| Cellulase/ Endo-β-1,4-glucanase | 2.03 | 26.1 |
| Exoglucanase | n.d. | 13.3 |
| Xyloglucanase | n.d. | 8.73 |
|  |  |  |
| *Esterases* |  |  |
|  |  |  |
| (4-*O*-methyl)-D-glucuronate—lignin esterase | n.d. | 8.68 |
| Carboxylic ester hydrolase | 2.07 | n.d. |
|  |  |  |
| *Transferases* |  |  |
|  |  |  |
| 1,3-β-Glucanosyltransferase | 3.92 | 0.21 |
|  |  |  |

**Table S2** EC numbers and CAZy families for enzyme activities above 2% relative expressions strength, enzyme activities detected in both A. awamori and Cellic CTec3 are shown in bold

| Enzyme name | EC number | CAZy family |
| --- | --- | --- |
| *Glucosidases* |  |  |
|  |  |  |
| Glucan-1,3-β-glucosidase | EC 3.2.1.58 | GH31; GH63 |
| Glucan-endo-1,3-β-D-glucosidase | EC 3.2.1.39 | GH10; GH11 |
| Glucoamylase | EC 3.2.1.3 | GH15; GH31 |
| α-Glucosidase | EC 3.2.1.20 | GH31 |
| β-Glucosidase | EC 3.2.1.21 | GH1; GH3 |
|  |  |  |
| *Mannosidases* |  |  |
|  |  |  |
| α-1,2-Mannosidase | EC 3.2.1.77 | GH5; GH55 |
|  |  |  |
| *Xylosidases* |  |  |
|  |  |  |
| Endo-1,4-β-xylanase | EC 3.2.1.8 | GH13; GH57 |
| Xylan-1,4-β-xylosidase | EC 3.2.1.37 | GH74; GH16 |
|  |  |  |
| *Arabinosidases* |  |  |
|  |  |  |
| Arabinan endo-1,5-α-L-arabinosidase | EC 3.2.1.99 | GH35 |
|  |  |  |
| *Chitinase/Hexosaminidases* |  |  |
|  |  |  |
| Chitinase | EC 3.2.1.14 | GH15; GH31 |
| β-Hexosaminidase | EC 3.2.1.52 | GH51; GH43; GH62 |
|  |  |  |
| *Cellulase/β-Glucanases* |  |  |
|  |  |  |
| Cellulase/ Endo-β-1,4-glucanase | EC 3.2.1.4 | GH5; GH7; GH9; GH12 |
| Exoglucanase | EC 3.2.1.91 | GH1; GH3 |
| Xyloglucanase | EC 3.2.1.151 | GH5; GH26 |
|  |  |  |
| *Esterases* |  |  |
|  |  |  |
| (4-*O*-methyl)-D-glucuronate—lignin esterase | EC 3.1.1.117 | CE15 |
| Carboxylic ester hydrolase | EC 3.1.1.1 | CE1; CE2; CE7 |
|  |  |  |
| *Transferases* |  |  |
|  |  |  |
| 1,3-β-Glucanosyltransferase | EC 2.4.1.34 | GT2; GT21; GT48 |
|  |  |  |

**Figure S8:** C. paradoxa 1% (w/v) algae plate uninoculated

**Figure S9**: C. paradoxa 1% (w/v) algae plate inoculated, 7 days at 28°C

**Figure S10:** A. awamori 1% (w/v) algae plate uninoculated

**Figure S11**: A. awamori 1% (w/v) algae plate inoculated, 7 days at 28°C

**Figure S12**: A. niger 1% (w/v) algae plate uninoculated

**Figure S13**: A. niger 1% (w/v) algae plate inoculated, 7 days at 28°C

**Figure S14**: C. paradoxa 5% (w/v), liquid media; inoculated; 7days, 28°C, 110 rpm

**Figure S15**: A. awamori 5% (w/v), liquid media; inoculated; 7days, 28°C, 110 rpm

**Figure S16**: A. niger , liquid media; inoculated; 7days, 28°C, 110 rpm
